# Enhanced magnetic Purcell effect in room-temperature masers

**DOI:** 10.1038/ncomms7215

**Published:** 2015-02-20

**Authors:** Jonathan Breeze, Ke-Jie Tan, Benjamin Richards, Juna Sathian, Mark Oxborrow, Neil McN Alford

**Affiliations:** 1 Department of Materials, Imperial College London, Royal School of Mines, Exhibition Road, London SW7 2AZ, UK

## Abstract

Recently, the world’s first room-temperature maser was demonstrated. The maser consisted of a sapphire ring housing a crystal of pentacene-doped *p*-terphenyl, pumped by a pulsed rhodamine-dye laser. Stimulated emission of microwaves was aided by the high quality factor and small magnetic mode volume of the maser cavity yet the peak optical pumping power was 1.4 kW. Here we report dramatic miniaturization and 2 orders of magnitude reduction in optical pumping power for a room-temperature maser by coupling a strontium titanate resonator with the spin-polarized population inversion provided by triplet states in an optically excited pentacene-doped *p*-terphenyl crystal. We observe maser emission in a thimble-sized resonator using a xenon flash lamp as an optical pump source with peak optical power of 70 W. This is a significant step towards the goal of continuous maser operation.

## Abstract (web summary)

Masers are promising for applications that use microwave radiation. Here, the authors present a compact room-temperature maser design using a high permittivity dielectric material for the resonator to achieve low optical pumping powers. This design pushes masers closer towards their promised applications.

## Graphical abstract

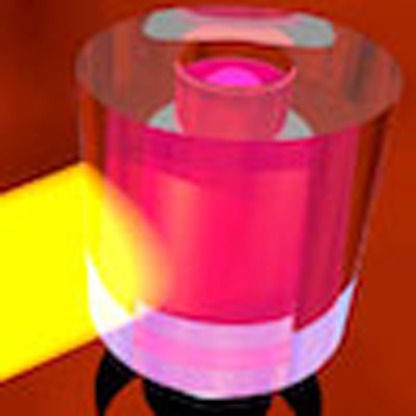

Historically, solid-state masers employing paramagnetic crystals have typically required large static magnetic fields to provide the necessary Zeeman splitting of the electronic spin states in addition to cryogenic cooling to reduce the detrimental effects of lattice vibrations on the lifetime of spin-polarized population inversions^1^,^2^. Efforts to avoid either cryogenic cooling or high magnetic fields have met with limited success. A zero magnetic field solid-state maser^3^ using a high quality factor (*Q*) whispering-gallery mode in a single crystal of sapphire with trace paramagnetic Fe^3+^ impurities was demonstrated a decade ago, but required cryogenic cooling to liquid helium temperature. The prospect of realizing a room-temperature maser using population inversions within photo-excited triplet-state sublevels in organic paramagnetic molecules was first proposed by Blank^4^,^5^,^6^ for a device operating in high magnetic fields. Although maser oscillation was not observed in the reported device, this work paved the way for the recent discovery of maser operation at room temperature and zero magnetic field^7^, which promises a new generation of miniaturized ultra-low-noise high-gain maser amplifiers and oscillators without the incumbent refrigeration and electromagnet infrastructure. Due to their compactness and simplicity, these devices could find application in research areas that rely on detection of weak microwave signals such as structural biology, space science and exploration, communications, quantum information processing and spectroscopy. Stimulated microwave emission at a frequency of 1.45 GHz was observed in a high *Q*-factor single-crystal sapphire ring housing a single crystal of pentacene-doped *p*-terphenyl. The pentacene molecules were optically excited from the *S*_0_ singlet ground state into *S*_1_ singlet states by a 350 μs duration pulsed rhodamine-dye laser with wavelength near 590 nm and peak optical power 1.4 kW (see Fig. 1). Electrons in the *S*_1_ state rapidly undergo spin-orbit coupling mediated intersystem crossing into the *T*_2_ triplet state with a spin-selective population of the X, Y and Z sublevels in the experimentally determined ratio of 0.76:0.16:0.08 (refs 8, 9). Electrons in the *T*_2_ triplet-state sublevels subsequently relax non-radiatively down into the *T*_1_ triplet state while preserving their spin polarization and resulting in a population inversion between the upper X and lower Z triplet sublevels. Spin-lattice relaxation processes can also occur between the triplet sublevels which eventually decay non-radiatively back to the ground state thus completing the cycle. At zero magnetic field, the frequency splitting between the X and Z triplet sublevels in pentacene-doped *p*-terphenyl is ~1.45 GHz, so if the spin-polarized paramagnetic molecules are situated within a suitable microwave resonator tuned to this frequency stimulated emission of microwaves due to transitions from the X to Z sublevels may result in amplification of the microwave photons present in the maser resonator if the rate of stimulated microwave emission is greater than the rate of decay due to losses in the cavity.

The sapphire-based maser operated in burst mode under pulsed optical illumination yet continuous maser operation is essential for a practically usable device. The threshold optical pump power (in pulsed mode) of the original device was estimated to be 230 W and although yellow lasers with such continuous-wave (CW) power output are not currently available, green single-mode fibre lasers are available that could provide the necessary power but they are large, complex and expensive. It seems that the room-temperature maser has replaced the system overhead of cryogenic operation with that of a high-power CW laser with its associated cooling systems and power supplies. However, since almost all the optical power absorbed by the pentacene molecules is transferred into heat, this places an enormous heat load burden on the gain medium, which has a low melting point of 213 °C. One way of relieving the burden of the heat load would be to find superior material systems that provide the necessary microwave paramagnetic gain while reducing heat dissipation. This is the subject of ongoing research in the field. In the meantime, it would also be advantageous to be able to realize room-temperature masers that can operate with reduced optical pumping power requirements. Here we describe a means to dramatically reduce the optical pumping requirement of a room-temperature Earth’s field maser by using a very high permittivity incipient ferroelectric. Two orders of magnitude reduction in the magnetic mode volume and a high *Q*-factor results in a miniaturized maser that can be optically pumped with a xenon flash lamp delivering peak optical power of 70 W. The estimated optical pump power threshold of this maser is 2 W.

## Results

### Optimizing the magnetic Purcell factor

For a maser based on organic paramagnetic triplet systems, the resonators in which they are placed must meet specific criteria for maser threshold conditions to be met. Stimulated transition rates between the triplet sublevels in a molecule such as pentacene are proportional to the magnetic energy density *μ*_0_|**H**|^2^ and spin–spin relaxation time (*T*_2_). The magnetic energy density can be shown to be inversely proportional to the magnetic mode volume (*V*_m_), a measure of the physical extent of the resonant mode. The fate of the microwave photons produced by stimulated emission is dictated by their lifetime, which is dependent on the resonator *Q*-factor. Higher *Q*-factors mean fewer microwave photons are absorbed by the dielectric and metallic materials from which the resonator is constructed. The ratio of *Q*-factor and magnetic mode volume was found by Purcell^10^, to provide an explanation for the enhanced spontaneous emission observed in radio-frequency magnetic resonance experiments. The Purcell effect enhances spontaneous and stimulated emission processes and is related to the local electromagnetic density-of-states.

The population dynamics of microwave photons and spin-polarized triplet sublevels within a maser can be modelled using coupled differential equations that govern the rate of change in the population of states. For steady-state conditions, a threshold equation for the optical power required to sustain maser oscillation can be derived for a simple two-level system (see Methods):


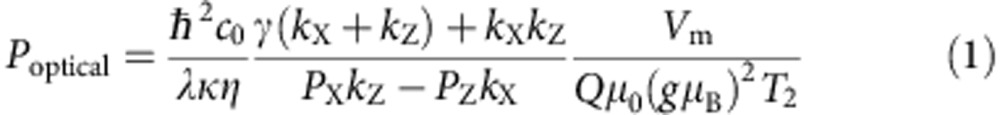


where *λ* is the optical pump wavelength, *κ* the optical coupling efficiency, *η* the intersystem crossing yield, *γ* the spin-lattice relaxation rate between the X and Z sublevels, *P*_X_ and *P*_Z_ the population ratios of the X and Z triplet-state sublevels (0.76 and 0.08, respectively) and *k*_X_, *k*_Z_ their decay rates back to the ground state *S*_0_. *V*_m_ is the magnetic mode volume, *Q* the cavity quality factor and *T*_2_ the spin–spin relaxation time for the X–Z transition. The optical power threshold is inversely proportional to the magnetic Purcell factor: *P*_optical_∝*V*_m_/*Q*∝1/*F*_m_. The magnetic Purcell factor itself can be optimized by increasing the *Q*-factor and minimizing the magnetic mode volume *V*_m_. Improvement of the *Q*-factor for a single-crystal sapphire-based resonator by at least an order of magnitude has been reported for a Bragg reflector resonator^11^, however, such resonators have larger mode volumes and are difficult to construct in practice. Alternatively, miniaturizing the maser resonator using high permittivity dielectric materials should reduce the mode volume since it scales as 
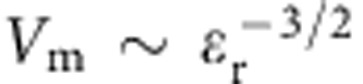
, where *ε*_r_ is the relative permittivity. This approach has been reported in the field of electron spin resonance spectroscopy^12^, where a potassium tantalate single crystal was successfully used to enhance the signal.

Incipient ferroelectrics are very interesting dielectric materials, exhibiting the highest relative permittivities of any non-piezoelectric dielectric. They are classed as incipient (almost) ferroelectrics since their relative permittivity increases dramatically as temperature decreases, suggesting that a ferroelectric phase transition is imminent. However, the phase transition temperature for incipient ferroelectrics is negative and so is not observed. In the case of quantum incipient ferroelectrics, the phase transition temperature is slightly positive but suppressed by zero-point quantum fluctuations. Their very high relative permittivity allows for substantial miniaturization of microwave resonators although their inherent high temperature coefficient of permittivity has excluded them from practical use. Indeed, the first demonstration of a dielectric resonator was constructed from single-crystal rutile but was deemed impractical due to its large temperature coefficient of permittivity^13^. A literature search was conducted to identify dielectric and incipient ferroelectric single crystals that had high relative electrical permittivity and low dielectric loss. Single crystals were chosen since their transparency permits optical excitation of the pentacene: *p*-terphenyl maser crystal. The candidate materials in addition to sapphire (Al_2_O_3_) were the incipient ferroelectrics rutile titanium dioxide (TiO_2_), potassium tantalate (KTaO_3_) and strontium titanate (SrTiO_3_ (STO))^14^,^15^. Maser resonators incorporating these materials were modelled and their dimensions were optimized to yield maximal Purcell enhancement factors for the TE_01*δ*_ mode resonant at the X↔Z zero-field-splitting frequency near 1.45 GHz. The TE_01*δ*_ mode is advantageous due to its cylindrical symmetry, high quality factor and magnetic field anti-node along its axis. The microwave magnetic field distribution in a TE_01*δ*_ mode resonator resembles a dipole with very strong axial magnetic field strength along the cylindrical axis. The results in Table 1 interestingly show that the magnetic Purcell factor enhancement for a STO maser resonator is over an order of magnitude greater than a sapphire maser resonator. Despite dielectric losses being high in incipient ferroelectrics such as STO, their very high relative permittivities mean that almost all electric and magnetic energy is contained within them when they form dielectric resonators, thus reducing ohmic losses in the metallic cavity walls to negligible levels.

### Experimental STO maser performance

**Figure 2:**
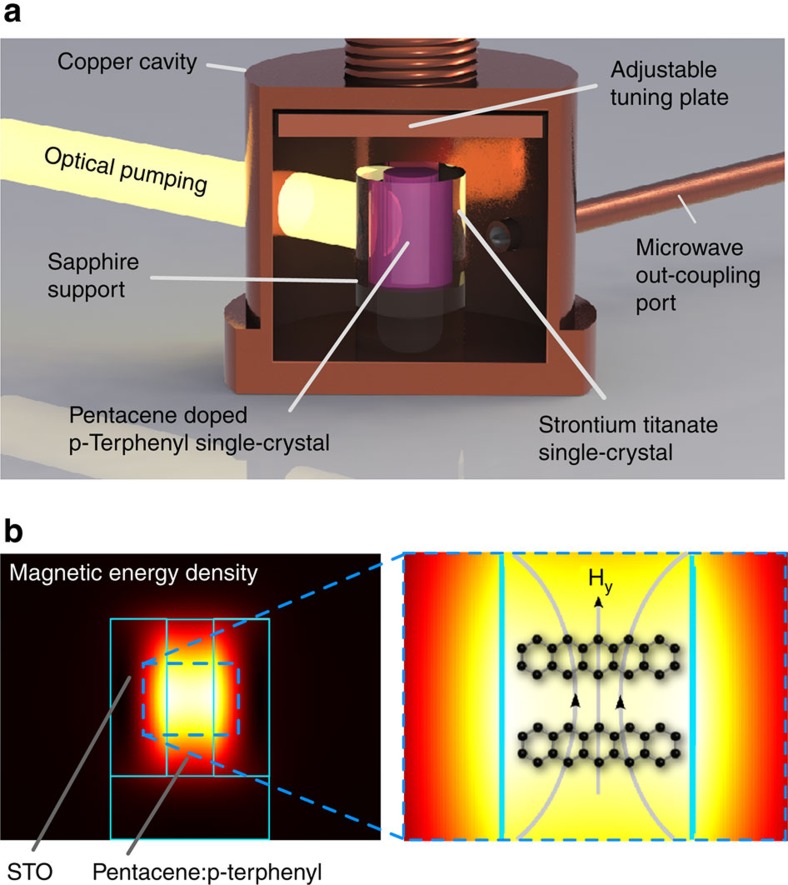
Strontium titanate maser construction. (**a**) Rendered image of strontium titanate (SrTiO_3_) maser. (**b**) Plot of magnetic energy-density distribution in the cross-section through maser displaying high concentration within the pentacene:*p*-terphenyl gain medium. The microwave magnetic field in the TE_01*δ*_ mode behaves like a magnetic dipole and induces X↔Z transitions in the pentacene triplet-state sublevels by having a magnetic field component along the molecular *y*-axes.

**Figure 3:**
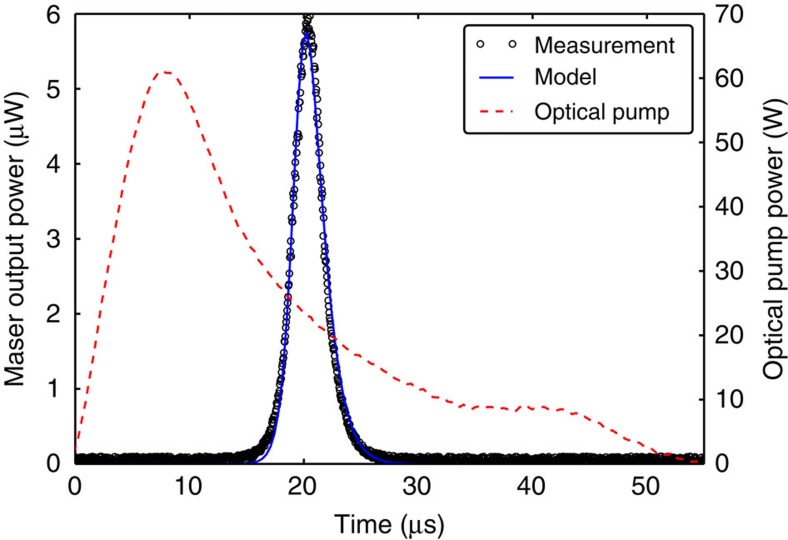
Temporal response of maser output power. Measured and modelled microwave burst from strontium titanate/pentacene:*p*-terphenyl maser excited by xenon flash lamp.

**Figure 4:**
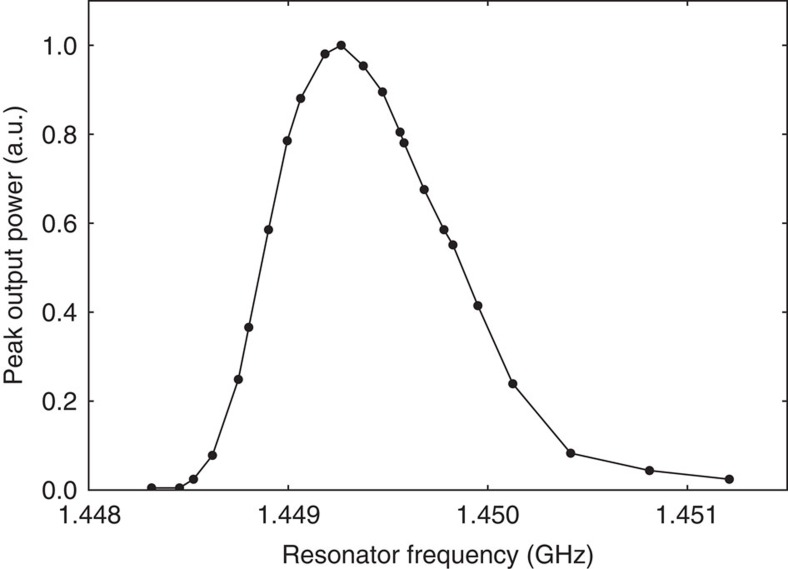
Frequency response of STO maser. The peak output power for different resonant frequencies of the TE_01*δ*_ mode, measured on illuminating the pentacene:*p*-terphenyl crystal within the strontium titanate resonator with a xenon flash lamp. The microwave power was coupled into an low-noise amplifier and detector and measured using a digital storage oscilloscope. The maximum peak power was observed at 1.4493 GHz.

A maser was constructed from a polished hollow cylinder of STO single crystal (SurfaceNet GmbH) grown by the flame fusion method. The STO cylinder enclosed a pentacene-doped *p*-terphenyl single crystal (grown in-house) within its bore as shown in Fig. 2 and aligned so that the molecular *y*-axes of the pentacene molecules had components along the cylindrical axis of the resonator, the same direction as the microwave magnetic field. This was done so that transitions between the X↔Z sublevels could be induced. The nested crystals were housed within a cylindrical copper cavity into which a microwave port (loop on the end of a coaxial cable) was introduced to couple out microwave power generated by stimulated emission. The resonant frequency of the maser could be manually tuned around the X↔Z zero-field transition frequency near 1.45 GHz. The maser output power coupling coefficient was measured using a second microwave port that was very weakly coupled to the maser cavity, allowing a two-port transmission measurement to yield the coupling coefficient of the weakly coupled port (−35 dB). The measured loaded *Q*-factor of the maser resonator including pentacene:*p*-terphenyl crystal without optical illumination was 8,900. A poly(methyl methacrylate) (PMMA) light pipe was bonded to the side of the STO resonator and protruded through an aperture in the cavity wall. Broadband optical pulses emitted from a xenon flash lamp (Perkin Elmer MVS 7010) at a repetition rate of 10 Hz were filtered to provide a spectrum in the visible range (450–650 nm) and focussed onto the light pipe which guided them into the cavity and onto the STO/pentacene:*p*-terphenyl resonator. Microwave power from the coupling port was amplified by a low-noise amplifier (22 dB gain) and fed into a microwave crystal detector (HP Agilent 420A) whose output voltage was recorded by a digital storage oscilloscope (Tektronix TBS 1022). The oscilloscope was triggered by a photodiode that was also used to measure the temporal power profile of the optical pulse emitted at the end of the light pipe near the maser resonator. Based on integrated-sphere measurements of the resonator, it was estimated that the peak optical power absorbed per pulse by the pentacene:*p*-terphenyl crystal was ~40 W with half-power pulse widths of ~10 μs. On illuminating the maser crystal with an optical pulse from the xenon flash tube, a strong maser burst of microwave power was observed ~20 μs after the onset of the optical pulse with a peak power of 6 μW (−22.2 dBm). The maser burst had a half-power temporal width of ~3 μs and on integration yielded an energy of 2.5 μJ, which corresponds to 2.6 × 10^13^ transitions between the X↔Z triplet sublevels accompanied by stimulated emission of microwave photons. Recorded time-domain optical pump and maser output power traces are shown in Fig. 3 along with a two-level rate equation model prediction of the maser output power which agrees well. The experiment was repeated for different resonant frequencies by manually tuning the maser cavity and recording the peak maser output power. The maser produced the strongest signal at 1.4493 GHz as shown in Fig. 4, which is assumed to be the actual X↔Z zero-field-splitting frequency for the pentacene: *p*-terphenyl crystal. The width of the frequency response of the maser (~900 kHz) is consistent with reported measurements^16^.

## Discussion

The time delay between the peak of the optical pulse and the microwave maser emission is due to the finite number of pentacene molecules in the gain medium and the *Q*-factor of the resonator. Growth in the microwave photon population within the maser mode (amplification) occurs when the rate of stimulated microwave emission exceeds the decay rate due to the losses within the resonator. The population inversion between the X and Z triplet sublevels is depleted until the peak of the maser burst is reached, where the rate of microwave photon production balances the rate of decay due to losses. Although the observed maser bursts only have a peak power of a few microwatts, they are the result of maser amplification of the number of thermal photons present in the cavity from a few thousand to over 10^13^. The total loss of the maser is given by the reciprocal of the loaded quality factor 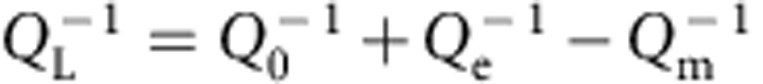, where *Q*_0_ is the unloaded quality factor of the maser resonator without optical illumination, *Q*_e_ is the external quality factor due to coupling losses and *Q*_m_ is the maser gain medium quality factor. The maser medium quality factor *Q*_m_ can be calculated during simulation, 
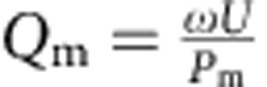
, where *U* is the energy stored in the maser mode and *P*_m_ is the stimulated emission power. From simulation, the average gain medium quality factor *Q*_m_ over the duration of the maser emission was calculated to be 4,500. If the maser medium gain, 
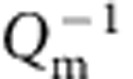
, is greater than the intrinsic cavity and coupling losses, 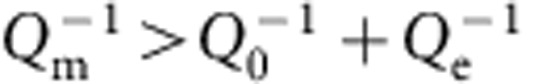, then the maser is configured as an oscillator and so the mode energy will grow until saturation or depletion of the inverted population. If the medium gain is greater than the intrinsic cavity losses but less than the total losses, 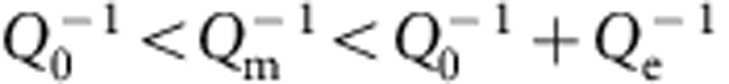, then the maser is configured as an amplifier with a bandwidth


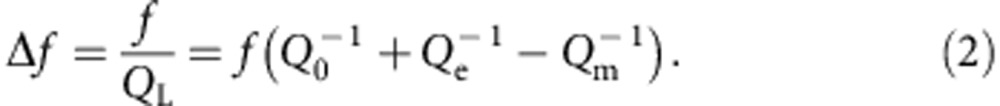


For a bandwidth of 1 MHz, which is approximately the width of the frequency response of maser emission as shown in Fig. 4, the external quality factor is required to be *Q*_e_=1,260. If the gain medium quality factor *Q*_m_ could be sustained continuously, then the power gain of such a maser when configured as an amplifier could be calculated as^2^:


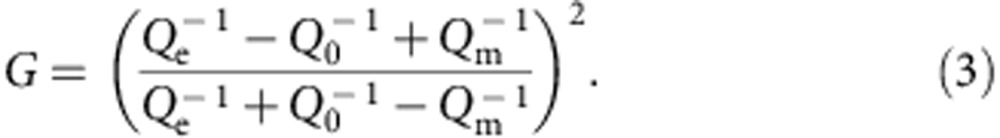


In this case the maser amplifier gain would be 2.7 dB, which although low could be improved by increasing the level of optical pumping power or reducing the bandwidth of the amplifier. For example, reducing the bandwidth to 0.1 MHz would yield a power gain of 12.1 dB.

Towards the goal of room-temperature masers being used as high-gain low-noise amplifiers in exciting and possibly new areas of research, here we have demonstrated a considerable step towards the practical realization of such a maser, by reducing the physical volume and optical pumping requirements by almost 2 orders of magnitude. The next step will be to achieve continuous maser operation and explore new organic molecular systems that could provide spin-polarized population-inverted triplet states with possibly more favourable characteristics.

## Methods

### Pentacene:*p*-terphenyl crystal growth

Commercially available pentacene powder (TCI Europe NV) was vacuum purified and *p*-terphenyl commercial powder (Alfa Aesar, 99+%, AL4833) was zone refined. About 0.006% mol per mol pentacene in *p*-terphenyl powder was prepared and sealed in a 3-mm inner diameter surface modified quartz ampoule with vacuum level of ~10^−3^ mbar. A sharp tip was made at one end of the ampoule for self-seeding. The wall surfaces of the ampoule were decorated with 1H,1H,2H,2H-perfluorodecyltrichlorosilane and cleaned thoroughly using solvents (acetone, anhydrous isooctane, isopropanol and distilled water) in an ultrasonic bath. A zone melting technique was used to grow the pentacene-doped *p*-terphenyl crystal. An in-house furnace’s temperature was controlled with a Eurotherm 3216 temperature controller and TE10A power controller to conduct the zone melting process at an elevated temperature of 200 °C. The melt zone temperature was set at 230 °C. The ampoule was lowered through the furnace at a rate of ~1 mm h^−1^ using a gear motor. Thereafter, the furnace was cooled down at 1 °C h^−1^ to room temperature and the ingot retrieved. The zone melting process swept pentacene guest molecules with the melt zone up to the top of the *p*-terphenyl ingot, leaving that region the most concentrated with pentacene. The top of the ingot with the highest pentacene concentration (determined from the most intense pink colour in the ingot) was selected for the experiments. Due to crystal growth habit, the triclinic *c*-plane exists along the ampoule long axis.

### Maser resonator design

The maser resonators were modelled using a quasi-analytical radial mode-matching technique^17^. For a given dielectric (Al_2_O_3_, TiO_2_, KTaO_3_ or STO) the geometries of the resonators were optimized to yield an optimal magnetic Purcell factor, given by *F*_m_=(4*π*/3*λ*^3^)*Q*/*V*_m_, where *Q* is the unloaded quality factor and *V*_m_ the magnetic mode volume for the fundamental TE_01*δ*_ mode. The unloaded *Q*-factor is the reciprocal of the sum of the losses within the cavity, such as ohmic losses in cavity walls and dielectric losses within the dielectric resonator and pentacene-doped gain medium. The loss tangent of pentacene-doped *p*-terphenyl was measured using a dielectric resonator technique and found to be very low (tan*δ*~10^−5^). A low electric filling factor (proportion of electric energy) of the gain medium (<1%) implies that the dielectric losses within the gain medium are almost negligible, confirming that the TE_01*δ*_ mode is an excellent choice for this application. The magnetic mode volume, *V*_m_, was calculated as the ratio of the stored magnetic energy within the cavity, *μ*_0_∫_V_|**H**(**r**)|^2^d*V*, to the maximum magnetic energy density, *μ*_0_|**H**(**r**)|^2^. Optimization of the resonator Purcell factors was accomplished using the Nelder–Mead simplex algorithm^18^.

### Maser population dynamics

The triplet-state sublevels X, Y and Z are populated by optical excitation of electrons from the ground state into excited singlet states, which then undergo spin-selective intersystem crossing into the *T*_2_ triplet state with population ratio 0.76:0.16:0.08 for X:Y:Z^8^,^9^. The maser mode at frequency *ω* is supported by a microwave cavity resonator. Without excitation and in thermal equilibrium with its surroundings, the cavity has a thermal population of ambient microwave photons with a mean photon number 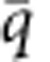 given by the Bose–Einstein occupation functions for bosons, 
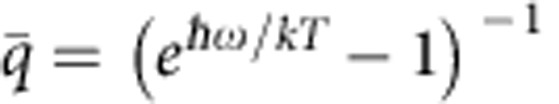
. The microwave photon number for the masing cavity mode is then given 
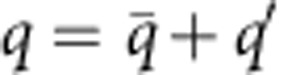
, where *q*′ represents the excess microwave photon number above the thermal level produced by stimulated emission. Spontaneous emission processes were not considered since they are negligible compared with stimulated processes at microwave frequencies. The cavity mode resonant frequency coincides with the splitting between the X and Z triplet manifold sublevels. The optical (pumping) photon power (photon flux) as a function of time is give by *p*(*t*), the optical coupling efficiency *κ* from the light pipe into the pentacene:*p*-terphenyl was estimated to be 50% and the intersystem crossing yield from the excited singlet to the triplet state (*S*_1_→*T*_2_), *η*_ISC_ is taken to be 62.5% at room temperature^19^. The rate equations governing the number of cavity photons and electrons in the ground state and triplet sublevels can be written:


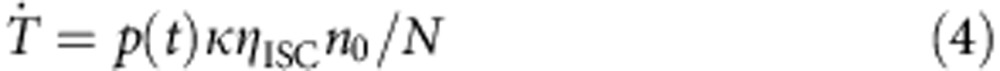



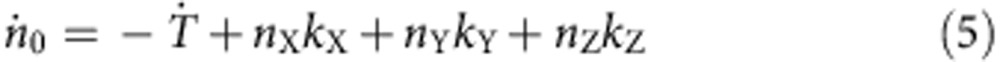



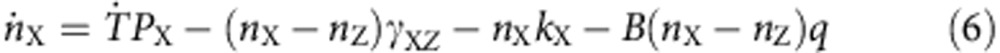



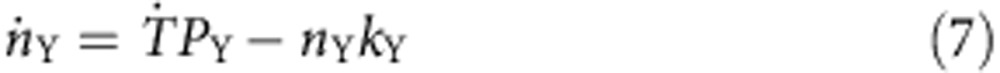



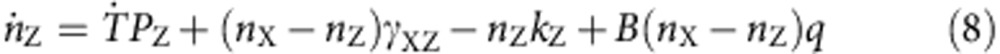



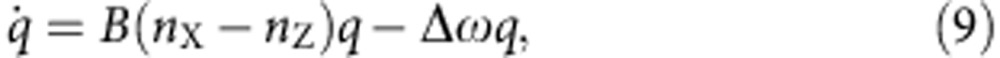


where *T* is the triplet population (sum of all X, Y and Z triplet sublevel populations); *n*_0_ is the population of pentacene molecules in the ground state (total available pentacene molecules *N*); *n*_X_, *n*_Y_ and *n*_Z_ are the populations of the X, Y and Z triplet sublevels; *P*_X_, *P*_Y_ and *P*_Z_ are the triplet sublevel population ratios (*P*_X_+*P*_Y_+*P*_Z_=1); *γ*_XZ_ is the spin-lattice relaxation rate between the X and Z sublevels and *k*_X_, *k*_Y_ and *k*_Z_ are the sublevel non-radiative decay rates back to the ground state. *B* is the Einstein coefficient for the transition rate of stimulated emission or absorption; *q* is the microwave photon population of the maser mode; Δ*ω* is the linewidth of the cavity mode, which is calculated from the loaded *Q*-factor, Δ*ω*=*ω*/*Q*_L_. Values for the spin-lattice relaxation rate, *γ*_XZ_, and triplet sublevel decay rates were taken from the literature^8^. The microwave photon power coupled out of the cavity at any time is given by *P*(*t*)=*q*(*t*)Δ*ωk*/(1+*k*), where *k* is the coupling coefficient of the microwave out-coupling port. The initial number of molecules in the *S*_0_ ground state, *n*_0_, is equal to the number of pentacene molecules *N*. Integration of the coupled differential rate equations used a standard solver for systems of coupled partial differential equations. The Einstein coefficient *B* was calculated from the the magnetic mode volume *V*_m_, the spin–spin relaxation time *T*_2_ was taken from ref. 20 and took into account the two different pentacene molecule orientations in the non-equivalent substitution sites in *p*-terphenyl at room temperature. A steady-state optical pump power threshold equation can be derived by disregarding the Y sublevel and setting the rate of change of cavity photons to zero, leading to:


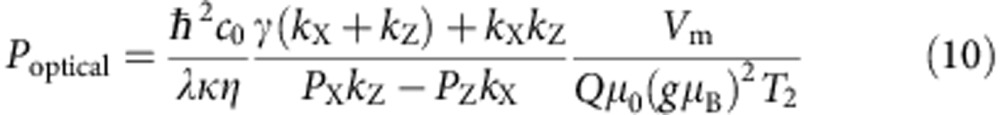


where *λ* and *κ* are the wavelength and coupling efficiency of the optical pump source, respectively, *V*_m_ is the magnetic mode volume and *Q* is the quality factor.

## Author contributions

J.B. performed calculations of enhanced magnetic Purcell factors in maser resonators using incipient ferroelectrics, designed the strontium titanate resonator and modelled the populations dynamics. K-J.T. grew the pentacene:*p*-terphenyl crystal. B.R. supported experimental set-up and microwave measurements of the maser. J.S. characterized the optical power delivery system. M.O. provided technical advice and guidance on aspects of the maser theory, design and experiment. J.B. and N.M.A. wrote the paper.

## Additional information

**How to cite this article**: Breeze, J. *et al*. Enhanced magnetic Purcell effect in room-temperature masers. *Nat. Commun.* 6:6215 doi: 10.1038/ncomms7215 (2015).

## Figures and Tables

**Figure 1:**
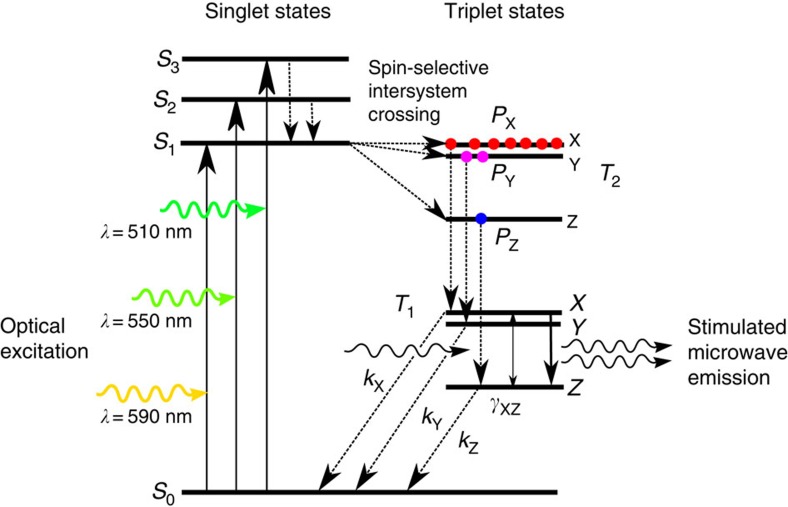
Maser process in pentacene. Jablonski diagram showing electronic state cycle responsible for generating population-inverted spin-polarized triplet-state sublevels in pentacene:*p*-terphenyl. Optical excitation of pentacene molecules from the ground state to the singlet states, *S*_0_→*S*_1_, *S*_2_, *S*_3_, followed by spin-orbit coupling mediated spin-selective intersystem crossing from the excited singlet states into the *T*_2_ triplet manifold. The *T*_2_ triplet states decay non-radiatively down to the *T*_1_ triplet state while preserving the spin-polarized populations. Spin-lattice relaxation can occur between the triplet sublevels with rate *γ*_XZ_. The triplet sublevels ultimately decay back to the *S*_0_ ground state with rates *k*_X,Y,Z_, thus completing a cycle. X→Z transitions due to stimulated emission lead to amplification of the microwave phonons in the maser cavity. Singlet excitation wavelengths were taken from optical extinction data^21^.

**Table 1:** Purcell factors of candidate masers.

**Dielectric**	***ε***_**r**_	***Q***	***V***_**m**_ **(cm**^**3**^**)**	***F***_**m**_
Al_2_O_3_	9	180,000	50.0	2.6 × 10^6^
TiO_2_	85	37,000	1.3	2.0 × 10^7^
KTaO_3_	241	12,500	0.3	3.0 × 10^7^
SrTiO_3_	318	10,000	0.2	3.6 × 10^7^

Modelled TE_01*δ*_ mode maser resonators with frequency 1.45 GHz incorporating single-crystal dielectrics of sapphire (Al_2_O_3_), rutile (TiO_2_), strontium titanate (SrTiO_3_) and potassium tantalate (KTaO_3_) with relative permittivity *ε*_r_, quality factor *Q*, magnetic mode volume *V*_m_ and magnetic Purcell enhancement *F*_m_. Each maser resonator was optimized to yield the maximal magnetic Purcell factor. The relative permittivity and loss tangents of TiO_2_, SrTiO_3_ and KTaO_3_ were taken from the literature^14^,^15^, the sapphire data from ref. 7.

## References

[b1] Bloembergen N. Solid state masers. Prog. Low Temp. Phys. 101, 396–429 (1961).

[b2] Siegman A. Microwave Solid-State Masers McGraw-Hill (1964).

[b3] Bourgeois P. . Maser oscillation in a whispering-gallery-mode microwave resonator. Appl. Phys. Lett. 87, 224104–224104 (2005).

[b4] Blank A., Kastner R. & Levanon H. Exploring new active materials for low-noise room-temperature microwave amplifiers and other devices. IEEE Trans. Microw. Theory Tech. 46, 2137–2144 (1998).

[b5] Blank A. & Levanon H. Applications of photoinduced electron spin polarization at room temperature to microwave technology. Appl. Phys. Lett. 79, 1694–1696 (2001).

[b6] Blank A. & Levanon H. Toward maser action at room temperature by triplet-radical interaction and its application to microwave technology. RIKEN Rev. 44, 128–130 (2002).

[b7] Oxborrow M., Breeze J. & Alford N. Room-temperature solid-state maser. Nature 488, 353–356 (2012). 22895341 10.1038/nature11339

[b8] Sloop D. J., Yu H., Lin T. & Weissman S. I. Electron spin echoes of a photoexcited triplet: pentacene in p-terphenyl crystals. J. Chem. Phys. 75, 3746–3757 (1981).

[b9] Lin T.-S. Electron spin echo spectroscopy of organic triplets. Chem. Rev. 84, 1–15 (1984).

[b10] Purcell E. Spontaneous emission probabilities at radio frequencies. Phys. Rev. 69, 681 (1946).

[b11] Breeze J., Oxborrow M. & Alford N. M. Better than Bragg: Optimizing the quality factor of resonators with aperiodic dielectric reflectors. Appl. Phys. Lett. 99, 113515 (2011).

[b12] Blank A., Stavitski E., Levanon H. & Gubaydullin F. Transparent miniature dielectric resonator for electron paramagnetic resonance experiments. Rev. Sci. Instrum. 74, 2853–2859 (2003).

[b13] Richtmyer R. D. Dielectric resonators. J. Appl. Phys. 10, 391–398 (1939).

[b14] Tobar M., Krupka J., Ivanov E. & Woode R. Anisotropic complex permittivity measurements of mono-crystalline rutile between 10 and 300K. J. Appl. Phys. 83, 1604–1609 (1998).

[b15] Geyer R., Riddle B., Krupka J. & Boatner L. Microwave dielectric properties of single-crystal quantum paraelectrics KTaO_3_ and SrTiO_3_ at cryogenic temperatures. J. Appl. Phys. 97, 104111 (2005).

[b16] Yang T.-C., Sloop D., Weissman S. & Lin T.-S. Zero-field magnetic resonance of the photo-excited triplet state of pentacene at room temperature. J. Chem. Phys. 113, 11194–11201 (2000).

[b17] Kajfez D. & Guillon P. Dielectric Resonators 1st edn Artech House Inc. (1964).

[b18] Nelder J. & Mead R. A simplex method for function minimization. Comput. J. 7, 308–313 (1965).

[b19] Takeda K., Takegoshi K. & Terao T. Zero-field electron spin resonance and theoretical studies of light penetration into single crystals and polycrystalline material doped with molecules photoexcitable to the triplet state via intersystem crossing. J. Chem. Phys. 117, 4940–4946 (2002).

[b20] Lang J., Sloop D. & Lin T.-S. Dynamics of p-terphenyl crystals at the phase transition temperature: a zero-field EPR study of the photoexcited triplet state of pentacene in p-terphenyl crystals. J. Phys. Chem. 111, 4731–4736 (2007). 10.1021/jp070251v 17497761

[b21] Nijegorodov N., Ramachandran V. & Winkoun D. The dependence of the absorption and fluorescence parameters, the intersystem crossing and internal conversion rate constants on the number of rings in polyacene molecules. Spectrochim. Acta A Mol. Biomol. Spectrosc. 53, 1813–1824 (1997).

